# Does Sleep Improve Memory Organization?

**DOI:** 10.3389/fnbeh.2014.00065

**Published:** 2014-04-08

**Authors:** Masashi Takeuchi, Hisakazu Furuta, Tomiki Sumiyoshi, Michio Suzuki, Yoko Ochiai, Munehito Hosokawa, Mie Matsui, Masayoshi Kurachi

**Affiliations:** ^1^Department of Neuropsychiatry, University of Toyama Graduate School of Medicine and Pharmaceutical Sciences, Toyama, Japan; ^2^Kamiichi General Hospital, Kamiichi-machi, Japan; ^3^Sleep Disorders Center, Hokuriku National Hospital, Nanto-shi, Japan; ^4^Department of Clinical Research Promotion, National Center Hospital, National Center of Neurology and Psychiatry, Tokyo, Japan; ^5^Department of Psychology, University of Toyama Graduate School of Medicine and Pharmaceutical Sciences, Toyama, Japan

**Keywords:** sleep, memory organization, Japanese Verbal Learning Test, wake after sleep onset, polysomnographic recordings

## Abstract

Sleep can integrate information into existing memory networks, look for common patterns and distil overarching rules, or simply stabilize and strengthen the memory exactly as it was learned. Recent research has shown that sleep facilitates abstraction of gist information as well as integration across multiple memories, insight into hidden solutions, and even the ability to make creative connections between distantly related ideas and concepts. To investigate the effect of sleep on memory organization, 35 normal volunteers were randomly assigned either to the sleep (*n* = 17) or wake group (*n* = 18). The sleep subjects performed the Japanese Verbal Learning Test (JVLT), a measure of learning and memory, three times in the evening, and slept. On the following morning (9 h later), they were asked to recall the words on the list. The wake subjects took the same test in the morning, and were asked to recall the words in the same time interval as in the sleep group. The semantic clustering ratio (SCR), divided by the total number of words recalled, was used as an index of memory organization. Our main interest was whether the sleep subjects elicit a greater increase in this measure from the third to the fourth assessments. Time × Group interaction effect on SCR was not significant between the sleep group and wake group as a whole. Meanwhile, the change in the SCR between the third and fourth trials was negatively correlated with duration of nocturnal waking in the sleep group, but not other sleep indices. Based on this observation, further analysis was conducted for subjects in the sleep group who awoke nocturnally for <60 min for comparison with the wake group. A significant Time × Group interaction was noted; these “good-sleepers” showed a significantly greater improvement in the memory index compared with the wake subjects. These results provide the first suggestion that sleep may enhance memory organization, which requires further study.

## Introduction

Sleep is important for a variety of physiological functions, e.g., homeostatic maintenance (Achermann and Borbély, 2005). Recently, sleep has been shown to enhance processing of information obtained during wakefulness, and consolidate it in the form of memory (Peigneux et al., 2001; Smith, 2001).

Memory is generally classified into short-term memory and long-term memory; the latter divided into episodic memory, semantic memory, and procedural memory (Tulving and Fergus, 2000). The concepts of memory organization refer to the possibility that the internal representation of a given perceptual input may assume different forms depending upon certain operation on the input, or on its representation in the memory store. Memory organization carries the implication of changes in the memory trace of an event that are influenced by the presence of certain other traces in the episodic memory store. Temporal encoding of an input implies the registration of the date of an episode without regard to other episodes, and it may not represent the way the system works, and the temporal date of a stored event may be determined by its organization in relation to other events with their temporal dates. Semantic encoding of a verbal item in episodic memory implies that the trace of the item is influenced by the information already available about its referent concept in semantic memory, while semantic organization refers to the grouping of items in a given set that reflects the semantic relations among the corresponding concepts. Memory organization refers to the collection and associations of memorized items and their recall (Kirimura, 1999), and implicitly serves as a strategy for facilitating memory itself. With memory organization, it is defined as “the method of summarizing, arranging, and memorizing related information.” Since other information which is related to it can be remembered as nature if more information can be memorized efficiently and certain information is remembered if information can be systematized by systematization, the burden in the case of search also decreases.

**Figure 1:**
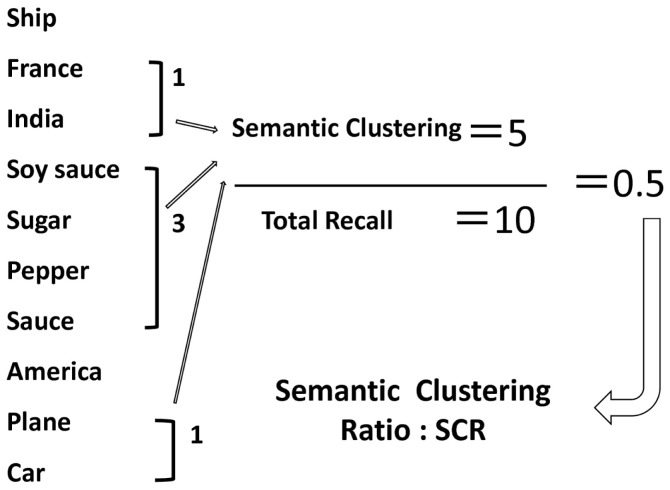
**An example of memory organization as assessed by the Japanese Verbal Learning Test**. The number of semantic categories (SCR) is counted when a separate word belonging to the same semantic category is recalled consequently. Thus, the SCR implicitly reflects the degree of memory organization. However, this index by itself can be affected by the total number of words recalled. Therefore, this study used the ratio of SCR vs. the total number of recalled words (SCR ratio) as an index of memory organization (see Materials and Methods).

From the view point of cognitive psychology, primary aspects of memory organization are already integrated in long-term memory. Generally, we unconsciously categorize items from the materials being learned, and judge novel items if they fall into a certain category. Memory organization is assessed by several ways, e.g., verbal fluency (Sumiyoshi et al., 2005), story memory (Sumiyoshi et al., 2001), and word memory (Nohara et al., 2000; Yamashita et al., 2000) tests. Accordingly, the Japanese Verbal Learning Test (JVLT), a word list test, was developed as a measure of memory organization (Yamashita et al., 2000). JVLT is a learning task and consists of a word list of 16 words. This test was developed based on previous reports (Gold, 1992) that employed three word lists (random, blocked, and unblocked lists), with a differential degree of semantic organization. The unblocked list is constituted by four semantic categories (animals, countries, musical instruments, and vegetables) with four words in each category. The unblocked list is organized in a manner that the words in the same category do not appear one after another. Thus, this list has an implicit category structure. JVLT uses a 16-word unblocked list involves four semantic categories with four words in each category. The words in the list are selected from everyday Japanese vocabulary; they are used approximately in the same frequency (Nohara et al., 2000; Yamashita et al., 2000). The number of semantic categories (semantic clustering ratio, SCR) is counted when a separate word belonging to the same semantic category is recalled consequently. Thus, the SCR implicitly reflects the degree of memory organization. However, this index by itself can be affected by the total number of words recalled. Therefore, this study used the ratio of SCR vs. the total number of recalled words (SCR ratio) as an index of memory organization (Matsui et al., 2006) (Figure 1). Further, this cognitive domain is impaired in patients with psychiatric diseases such as schizophrenia (Nohara et al., 2000).

A relationship between memory and sleep has been reported (Stickgold, 2005). For example, a facilitative effect of sleep on declarative memory has been demonstrated in humans (Stickgold and Walker, 2007). Specifically, performance on paired-association task has been associated with slow-wave sleep (Plihal and Born, 1997; Gais et al., 2002; Gais and Born, 2004), REM sleep (Koninck et al., 1989), stage 2 sleep (Walker et al., 2002), and spindles (Fogel et al., 2007). In particular, subjects with sleep apnea syndrome perform worse on tests of memory consolidation compared with healthy individuals (Kloepfer et al., 2009). Further, sleep deprivation has been shown to impair attention span (Hsieh et al., 2010). These lines of evidence suggest an importance of an appropriate amount of sleep in general and the maintenance, and, possibly, enhancement of memory.

The over lapping replay of related memories selectively strengthens shared elements (Lewis and Durrant, 2011). Repeated reactivation of memories in different combinations progressively builds schematic representations of the relationships between stimuli. Sleep can integrate information into existing memory networks, look for common patterns and distil overarching rules, or simply stabilize and strengthen the memory exactly as it was learned (Stickgold and Walker, 2013).

The above considerations led us to hypothesize that sleep would improve memory organization. To date, however, there is little information on the link between this domain of cognitive ability and sleep. Therefore, the present study was performed to determine whether organization of memory, as evaluated word list learning, would be enhanced during sleep in healthy subjects.

## Materials and Methods

### Subjects

Thirty-five healthy female subjects (university students; average age: 20.9 ± 1.9 years) participated in the study. They were randomly assigned into either the sleep group (*n* = 17) or wake group (*n* = 18) (Table 1). All participants were right-handed, had an academic history of 12 years, and were without physical problems (head trauma) or mental health problems (schizophrenia, mood disorder, dependency syndrome). No subjects were receiving medical treatment or suspected of having sleep disorders as determined by the International Classification of Sleep Disorders, Second Edition (American Academy of Sleep Medicine, 2005). All subjects were given an explanation of the research, including the instruction that they can withdraw from the study at any time. Subjects were paid for their participation in this study, and written informed consent was obtained.

**Table 1:** **Demographic data**.

	Sleep group (*n* = 17)	Wake group (*n* = 18)
Age (years)	20.4 ± 0.5	21.3 ± 2.5
Education (years)	12.0 ± 0.0	12.0 ± 0.0
IQ	99.6 ± 8.0	99.5 ± 8.6
JVLT1 total reproduction	11.4 ± 2.1	10.9 ± 1.7
SCR(1) ratio	0.31 ± 0.2	0.42 ± 0.2

*JVLT, Japanese Verbal Learning Test; SCR, semantic clustering ratio. Mean ± SD*.

### Japanese Verbal Learning Test

Japanese Verbal Learning Test is a learning task and consists of a word list of 16 words (Matsui et al., 2007a). The list is constituted by four semantic categories (animals, countries, musical instruments, and vegetables) with four words in each category. The list is organized in a manner that the words in the same category do not appear one after another. Thus, this list has an implicit category structure. JVLT uses a 16-word unblocked list involves four semantic categories with four words in each category. The words in the list are selected from everyday Japanese vocabulary; they are used approximately in the same frequency (Nohara et al., 2000; Yamashita et al., 2000).

The number of semantic categories (SCR) is counted when a separate word belonging to the same semantic category is recalled consequently. Thus, the SCR implicitly reflects the degree of memory organization. However, this index by itself can be affected by the total number of words recalled. Therefore, this study used the ratio of SCR vs. the total number of recalled words (SCR ratio) as an index of memory organization (Matsui et al., 2006) (Figure 1).

The Japanese Adult Reading Test (JART) was used to measure IQ. Subjects are required to read aloud 100 *kanji* (Chinese character) idioms (Matsuoka et al., 2002). IQ is calculated as follows; estimated IQ = 126.5 − 0.72*x* (the number of wrong answers).

### Polysomnographic recordings

Polysomnography was performed based on standardized techniques (Rechtschaffen and Kales, 1968). Digital electroencephalography (EEG), electromyography (EMG), and electrooculography (EOG) signals were acquired by Polymate system (DIGITEX LAB.CO., LTD, Tokyo, Japan). EEG electrodes (Fp1, Fp2, C3, C4, P3, P4, O1, O2) were placed on the subject’s head according to the International 10-20 system. Parameters for EEG recordings were as follows: sampling rate – 200 Hz, low- and high-pass filter – 0.3 and 60 Hz, and notch filter – 50 Hz. Polysomnography (PSG) data were scored automatically by Night Owl Professional (NoruPro Light Systems, Inc., Tokyo, Japan), and were rescored visually every 30-s epoch, according to standardized techniques (Rechtschaffen and Kales, 1968).

### Procedure

**Figure 2:**
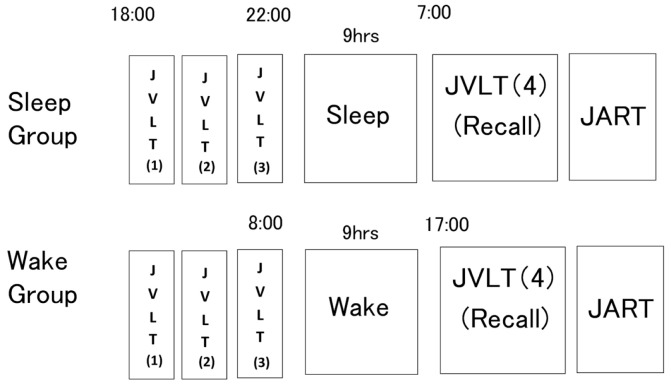
**Experimental procedure**. The sleep group received a learning session by means of three consecutive administrations of the Japanese Verbal Learning Test (JVLT) before initiation of sleep. On the morning of the following day, subjects were asked to recall as many words as possible without presentation of words. The wake group underwent the same examination procedure, as described in the figure (below).

Figure 2 shows the experiment procedure. The sleep group was instructed to report to the laboratory at 18:00. They took the JVLT. In the JVLT, subjects were instructed to remember as many words as possible without knowing the existence of any category. JVLT was consecutively performed three times before subjects were allowed to sleep. Each word was orally presented by the examiner with 1-s intervals. After all words had been presented, subjects were asked to recall them immediately. They were instructed to sleep. At the following morning, the fourth JVLT was performed. For this trial, subjects were asked to recall the words once without presentation of the stimulus words. This testing had not been notified to the subjects at the time of completion of the third JVLT. After took the fourth JVLT, subjects took the JART.

The wake group performed the same examination during waking hours with the same 9-h interval between the third and fourth trials of the JVLT as in the sleep group (08:00 and 17:00). These subjects were instructed not to perform excessive exercise or sleep during the interval. Adherence to these directions was confirmed with an Actigraph.

### Statistical analysis

Mann–Whitney test was used to compare age, academic background (year), estimated IQ, and the change of SCR ratio between the sleep and wake groups. Data from the total number of words recalled and SCR ratio were examined using repeated measures analysis of variance (ANOVA) with Group (wake group vs. sleep group) or (wake group vs. good sleep group) as between subject variable, and time (time points for the third and fourth JVLT administrations) as within-subject variable.

A *t*-test whether the two groups (wake group and good sleep group) indeed significantly differed before the retention interval between time point 3 and 4. A repeated measures analysis of covariance (ANCOVA) was performed for the memory organization parameter at time point 3 as covariate in the analysis of the change in memory organization between time point 3 and 4. Correlations between the change in the SCR ratio and sleep variables [total sleep time (TST), time in stage 1, time in stage 2, time in stage 3, time in stage 4, time in stage 3 and 4, time in REM sleep, time in non-REM sleep, and wake after sleep onset (WASO)] and wake variables were analyzed using Spearman’s rank correlation coefficient test.

## Results

Data from one subject in the sleep group were excluded from the analysis due to considerable artifacts in the polysomnography data. There were no significant differences between the sleep and wake groups in terms of age, academic background, and IQ (Table 1). The quality of sleep for the current subjects was the following results: stage 1 (67.8 ± 16.3 min), stage 2 (250.8 ± 22.6 min), slow-wave sleep (stage 3 + stage 4) (82.9 ± 14.3 min), REM sleep (74.2 ± 17.4 min), WASO (52.1 ± 23.4 min), and TST (480.3 ± 19.2 min).

**Figure 3:**
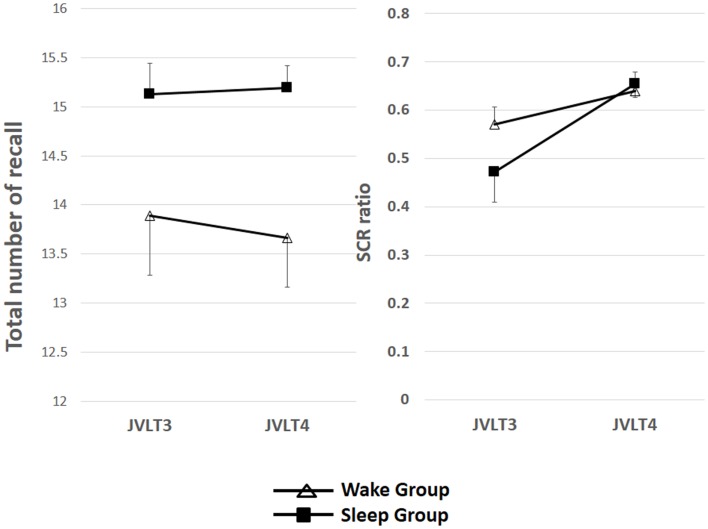
**The total number of words recalled (left) and the SCR ratio (right) from the JVLT test in awake (*n* = 18) and sleep (*n* = 17) groups**. Time × Group interaction effect was not significant for the total number of words recalled [*F*(1, 32) = 0.26, *p* = 0.61], and a significant main effect of time was not observed [*F*(1, 32) = 0.82, *p* = 0.78]. Time × Group interaction was not significant for the SCR ratio [*F*(1, 32) = 2.77, *p* = 0.11], although a significant main effect of Time was observed [*F*(1, 32) = 13.2, *p* = 0.01]. The error bars represent standard error of the mean.

The total number of words recalled and SCR ratio are shown in Table 2 and Figure 3.

**Table 2:** **Demographic data**.

	Sleep group		Wake group	
	Total number of recall	SCR ratio	Total number of recall	SCR ratio
JVLT1	11.4 ± 0.5	0.31 ± 0.05	10.9 ± 0.4	0.42 ± 0.03
JVLT2	13.9 ± 0.4	0.41 ± 0.05	13.4 ± 0.6	0.37 ± 0.04
JVLT3	15.1 ± 0.3	0.47 ± 0.06	13.9 ± 0.6	0.57 ± 0.04
JVLT4	15.2 ± 0.2	0.66 ± 0.03	13.7 ± 0.5	0.64 ± 0.04

*Mean ± SE*.

Time × Group interaction effect was not significant for the total number of words recalled [*F*(1, 32) = 0.26, *p* = 0.61], and a significant main effect of time was not observed [*F*(1, 32) = 0.82, *p* = 0.78]. Time × Group interaction was not significant for the SCR ratio [*F*(1, 32) = 2.77, *p* = 0.11], although a significant main effect of time was observed [*F*(1, 32) = 13.2, *p* = 0.01].

**Figure 4:**
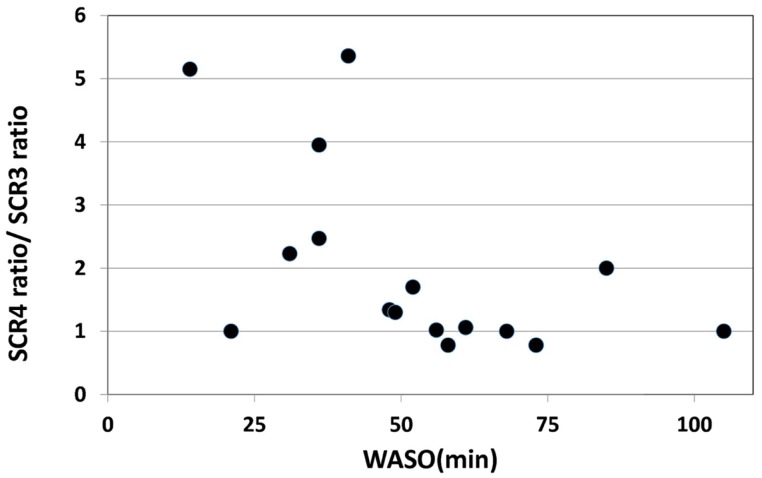
**Correlation between the change in the SCR ratio and the amount of nocturnal waking, as represented by WASO (wake after sleep onset) in the sleep group (*r*_s_ = 0.53,*p* < 0.05)**.

**Figure 5:**
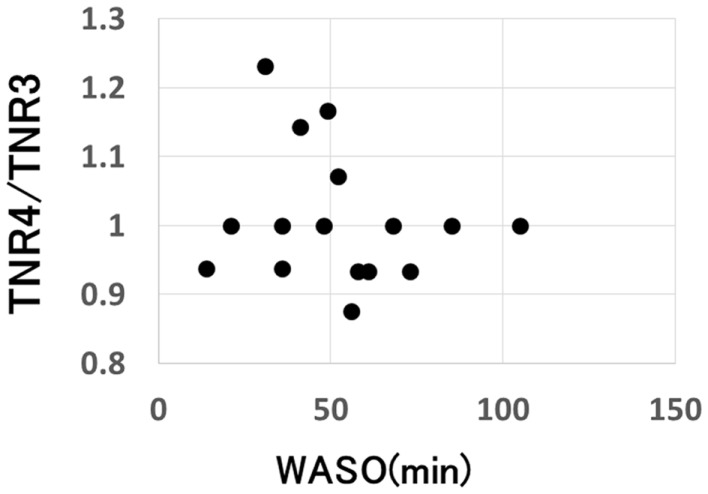
**Correlation between the change in the total number of recall and the amount of nocturnal waking, as represented by WASO (wake after sleep onset) in the sleep group (*p* = 0.31)**.

On the other hand, a significant negative correlation was observed between the change of SCR ratio from the third to fourth trials and nocturnal waking, as represented by WASO (*p* = 0.023, *r*_s_ = −0.53) (Figure 4), but no correlation was observed between the change of total number of recall (TNR) from the third to fourth trials and nocturnal waking (*p* = 0.31, *r*_s_ = −0.27) (Figure 5).

In view of these results, further analysis was conducted to determine if subjects with a small amount of nocturnal waking (WASO ≤ 60 min) would elicit a greater improvement of memory organization compared with awake subjects. It is said that wake after sleep onset is 75–80% of total sleep time (Hori, 2006). In our subjects, 75–80% of total sleep time is about 30 min. So we distinguished wake after sleep onset by being twice of 30 min.

**Figure 6:**
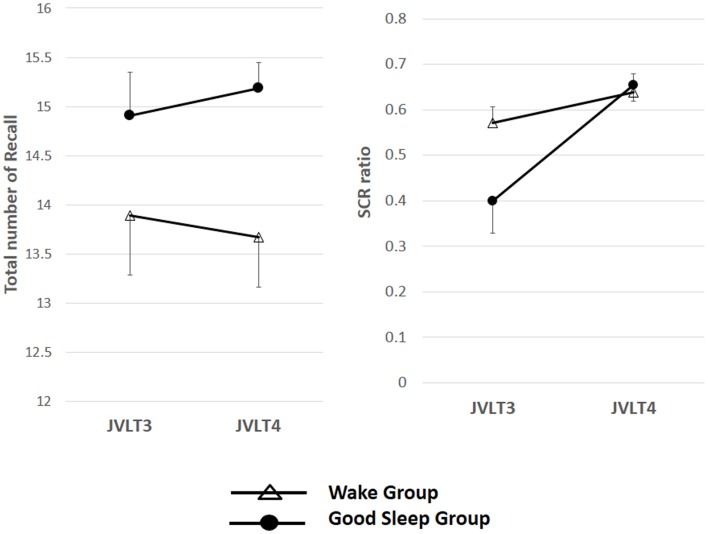
**The total number of words recalled (left) and the SCR ratio (right) from the JVLT in the good sleeper group (*n* = 11) and control subjects (*n* = 15)**. Left; repeated measures ANOVA was conducted to see the effect of sleep on the total number of recall with Group [sleepers showing WASO ≤60 min (*n* = 11) vs. awake subjects] as between subject variable, and time as within-subject variable. Time × Group interaction effect was not significant for the total number of words recalled [*F*(1, 27) = 0.57, *p* = 0.46], and a significant main effect of Time was not observed [*F*(1, 27) = 0.06, *p* = 0.94]. The error bars represent standard error of the mean. Repeated measures ANOVA was conducted to see the effect of sleep on the SCR ratio with Group [sleepers showing WASO ≤60 min (*n* = 11) vs. awake subjects] as between subject variable, and time as within-subject variable. A significant Group × Time interaction was observed in the SCR ratio [*F*(1, 27) = 6.23, *p* = 0.019].

Repeated measures ANOVA was conducted to see the effect of sleep on the SCR ratio with Group [sleepers showing WASO ≤60 min (*n* = 11) vs. awake subjects] as between subject variable, and time as within-subject variable. A significant Group × Time interaction was observed in the SCR ratio [*F*(1, 27) = 6.23, *p* = 0.019] (Figure 6).

On the other hand, repeated measures ANOVA was conducted to see the effect of sleep on the TNR with Group [sleepers showing WASO ≤60 min (*n* = 11) vs. awake subjects] as between subject variable, and time as within-subject variable. Time × Group interaction effect was not significant for the total number of words recalled [*F*(1, 27) = 0.57, *p* = 0.46], and a significant main effect of time was not observed [*F*(1, 27) = 0.06, *p* = 0.94].

**Figure 7:**
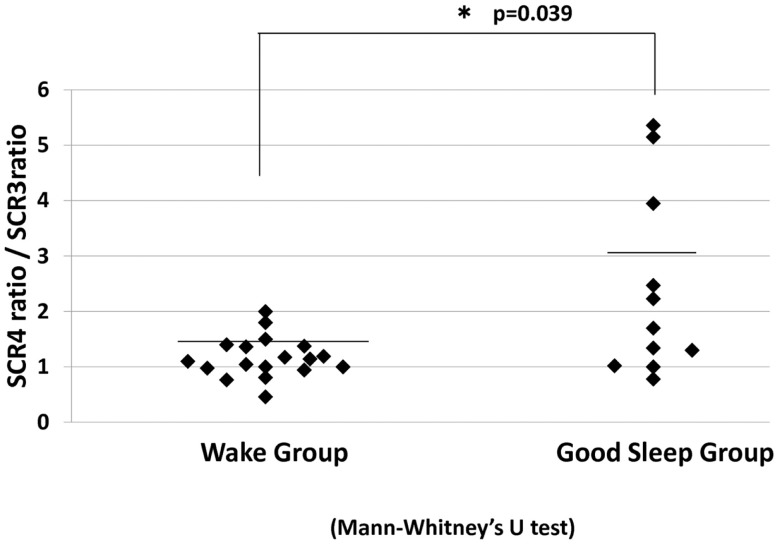
**SCR ratio for the fourth JVLT trial/SCR ratio for the third trial for the wake group and good sleep groups**. *p < 0.05 by Mann–Whitney *U*-test.

Subsequent analysis indicated a significant increase in the SCR ratio from the third to fourth JVLT trials only in the sleep group (*p* < 0.01). In fact, the change in the SCR ratio was significantly greater for these “good-sleepers” compared to wake subjects (Mann–Whitney test, *U* = 53, *p* = 0.04) (Figure 7).

When using a *t*-test to see whether the two groups (wake group and good sleep group) almost significantly differed before the retention interval between time point 3 and time point 4, there was a significant different between two groups [*F*(1, 27) = 4.34, *p* = 0.047].

A repeated measures ANCOVA was performed for the memory organization parameter at time point 3 as covariate in the analysis of the change in memory organization between time point 3 and 4, there was no significant difference at time point 3 and 4 [*F*(1, 27) = 1.12, *p* = 0.32]. Although no significant main effect of Group was observed [*F*(1, 27) = 2.084 *p* = 0.35], significant main effect of the Time was observed [*F*(1, 27) = 18.30 *p* < 0.01], and significant Group × Time interaction was observed [*F*(1, 27) = 6.23, *p* = 0.02].

**Figure 8:**
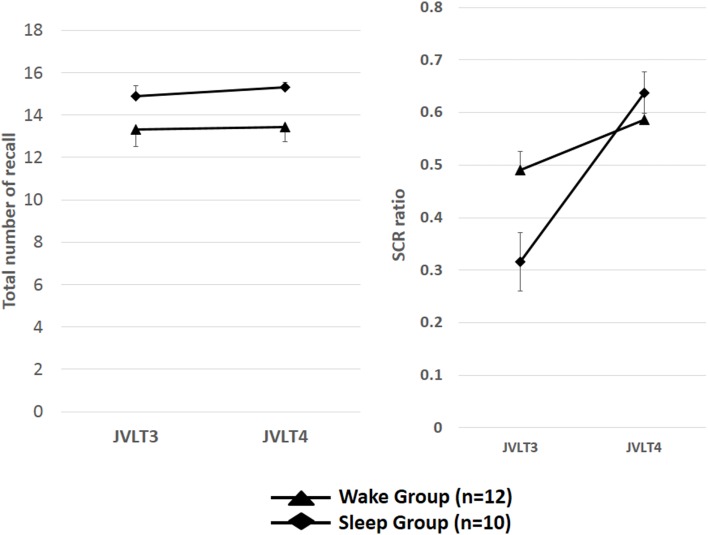
**The total number of words recalled (left) and the SCR ratio (right) from the JVLT in the SCR ratio in the third JVLT under 0.65 (*n* = 10) and awake subjects showing the SCR ratio in the third JVLT under 0.65 (*n* = 12)**. A significant Group × Time interaction was observed in the SCR ratio [*F*(1, 20) = 4.25, *p* = 0.01]. Subsequent analysis indicated a significant increase in the SCR ratio from the third to fourth JVLT trials only in the sleep group (*p* < 0.05), but not awake group (*p* = 0.57). The error bars represent standard error of the mean.

In comparison of the index of organization with the good sleep group and the awake group, the value of SCR ratio in the third JVLT was close to the maximum (0.75) by the awake group. Therefore, it was also considered that a difference did not appear in the SCR ratio in the third JVLT and the SCR ratio in the fourth JVLT in the awake group according to the ceiling effect of subject results. Therefore, after carrying out a group division by whether the SCR ratio in the third JVLT is 0.65 or more, the awake group and the sleep group conducted same analysis again, and examined whether a difference would come out of both groups. Repeated measures ANOVA was conducted to see the effect of sleep on the SCR ratio with Group [sleepers showing the SCR ratio in the third JVLT under 0.65 (*n* = 10) vs. awake subjects showing the SCR ratio in the third JVLT under 0.65 (*n* = 12)] as between subject variable, and Time as within-subject variable. A significant Group × Time interaction was observed in the SCR ratio [*F*(1, 20) = 4.25, *p* = 0.01]. Subsequent analysis indicated a significant increase in the SCR ratio from the third to fourth JVLT trials only in the sleep group (*p* < 0.05), but not awake group (*p* = 0.57) (Figure 8).

## Discussion

In the sleep group, a significant negative correlation was observed between the changes of memory organization index wake after sleep onset. We compared memory organization between sleep and wake group, but found no significant difference between the groups. So, we needed more subanalyses. In sub analyze, sleep subjects elicited an increase in memory organization compared with awake subjects. On the other hand, sleep did not affect the number of recalled words. To our knowledge, these findings indicate that sleep may enhance memory organization.

A prominent theory proposes that three characteristic field potentials, “slow oscillations,” “spindles,” and “sharp” wave-ripples are involved in some forms of sleep-dependent consolidation (Lewis and Durrant, 2011). During SWS, memories newly encoded into a temporary store (i.e., the hippocampus in the declarative memory system) are repeatedly reactivated, which drives their gradual redistribution to the long-term store (i.e., the neocortex). System consolidation during SWS relies on a dialog between neocortex and hippocampus under top-down control by the neocortical slow oscillations. The depolarizing up phases of the slow oscillations drive the repeated reactivation of hippocampal memory representations together with sharp wave-ripples and thalamo-cortical spindles. This synchronous drive allows for the formation of spindle-ripple events where sharp wave-ripples and associated reactivated memory information become nested into succeeding troughs of a spindle (Rasch and Born, 2013).

The neural basis for memory formation has been investigated by using several modalities. In a positron emission tomography study (Fletcher et al., 1998), performance on a task to generate an organization structure in a word list was associated with activation in the left prefrontal cortex in normal subjects. Functional magnetic resonance imaging demonstrated the left inferior prefrontal cortex elicits increased activation during semantic encoding (Demb et al., 1995). Furthermore, a recent study using near-infrared spectroscopy showed activation of prefrontal cortex during a memory organization task in healthy people (Matsui et al., 2007b). These findings suggest the role for prefrontal cortex in memory performance and its organization.

Suppression of cholinergic activity during SWS alleviates tonic inhibition of hippocampal CA3 and CA1 feedback neurons, thereby it enables spontaneous reactivations of the hippocampal networks and of the memory information encoded in these networks, as well as the transfer of the reactivated information to neocortical networks (Rasch and Born, 2013).

The acetylcholine system in the hippocampus has been suggested to regulate the acquisition of new knowledge (Terry and Buccafusco, 2003). For example, cholinergic activity is low during slow-wave sleep (Gais and Born, 2004) that improves memory performance, as mentioned above. Specifically, it is assumed that the direction of cholinergic activity regulate type of sleep, which affects declarative memory. For example, Gais and Born (2004) proposed an antagonistic relationship between the acetylcholine system and memory formation, i.e., cholinergic transmissions increase during waking or REM sleep, while they are reduced during slow-wave sleep. Taken together, acetylcholine is considered a major neurochemical substance for all stages of sleep, and affects some types of cognitive abilities.

Other neurotransmitters may also play a role in the ability of sleep to enhance memory. For example, Jouvet (1972) proposed monoamines (noradrenaline, serotonin) are also involved in the regulation of non-REM sleep (Jouvet, 1972). Further, REM sleep has been reported to be regulated by monoamines, as well as by acetylcholine (Hobson et al., 1975). These observations suggest neurotransmitters affect memory performance by modulating the quality of sleep.

Results in the present study suggest nocturnal waking is disadvantageous for memory organization, suggesting the importance of the quality of sleep. Specifically, sleep selectively improved an index of memory organization but not total words recalled, a measure of learning memory itself. The reason for this discrepancy may include that the number of words in the JVLT did not pose sufficient workload. The easiness of memory tasks have been reported to make it difficult to interpret data regarding the effect of sleep (Empson and Clark, 1970). In fact, our findings indicate a ceiling effect in both groups for the total number of words recalled in the third JVLT [Figure 3 (left) and Figure 6 (left)]. On the other hand, the workload for memory organization may have been appropriate to assess the effect of sleep, as implicated in Figure 3 (right) and Figure 6 (right). It considered about the influence of the ceiling effect of a memory organization task, only the sleep subjects obtained the result that organization was promoted by sleep, to the result of having divided and examined the group again (Figure 8).

It is possible that the greater hike of the SCR ratio in good sleeper subjects compared to controls may have been a result of the ability of the former subjects to effectively restore semantic knowledge. Alternatively, it is also assumed that the ability to organize memory was enhanced by sleep. Recent research has shown that sleep facilitates abstraction of gist information as well as integration across multiple memories, insight into hidden solutions, and even the ability to make creative connections between distantly related ideas and concepts (Lewis and Durrant, 2011; Griessenberger et al., 2012). Our findings may also suggest these findings.

Abnormalities in the sleep structure, such as the decreased slow-wave sleep and increased nocturnal waking, have been reported in patients with depression (Tsuno et al., 2005) or schizophrenia (Zarcone et al., 1998). It is also reported that sleep-dependent memory consolidation is absent or weakened in people suffering from conditions that cause sleep disturbances. For example, consolidation of declarative memory is absent in patients with sleep apnea syndrome (Kloepfer et al., 2009). These observations suggest that adequate sleep may be effective in enhancing some types of cognitive functions, such as organizing of information, in clinical subjects.

We compared memory organization between sleep and wake group, but found no significant difference between the groups. So, we needed more subanalyses.

The difference between groups before the retention interval should be considered as a major limitation, and the fact that ceiling performance was reached for total word recall as well as for the memory organization parameter in the wake group. The absence of a significant relationship between memory organization and other sleep variables, e.g. amount of specific stages of sleep, could be due to the small sample size. We need more analyze another sleep index. In addition, the quality of sleep is much worse as in other sleep studies including young and healthy participants (Carskadon, 2005; Payne et al., 2012). This is thought that we did not set up adaptation night, so the sleep subjects would not sleep well. The sleep and the wake group would effect by circadian rhythm on learning. Further controlled study with a larger number of subjects and control procedure controlled circadian rhythm effect is warranted to investigate the role for sleep in memory and other cognitive abilities.

## Conflict of Interest Statement

The authors declare that the research was conducted in the absence of any commercial or financial relationships that could be construed as a potential conflict of interest.
